# MoCloFlex: A Modular Yet Flexible Cloning System

**DOI:** 10.3389/fbioe.2019.00271

**Published:** 2019-10-17

**Authors:** Carlo A. Klein, Leonie Emde, Aaron Kuijpers, Patrick Sobetzko

**Affiliations:** ^1^SYNMIKRO, LOEWE Center for Synthetic Microbiology, Philipps-Universität Marburg, Marburg, Germany; ^2^Department of Biology, University of Kassel, Kassel, Germany

**Keywords:** cloning, golden-gate, DNA assembly, synthetic biology, gene arrangement, MoClo

## Abstract

Modern cloning solutions are gradually replacing classical cloning methods. Current systems make use of libraries with predefined DNA parts that are joined by Golden-Gate reactions. However, these systems still suffer from specific inflexibilities and the lack of inter-compatibility. Here, we present Flexible Modular Cloning (MoCloFlex) which overcomes this inflexibility by introducing a set of linker- and position-vectors allowing free unit arrangement. Our system, therefore, provides a convenient way to design and build custom plasmids, and iterative assembly of large constructs. To support standardization in synthetic biology, MoCloFlex is compatible with the well-established Modular Cloning standard. Here, we present and characterize MoCloFlex for various applications with up to 12 fragments in a single restriction-ligation reaction.

**Graphical Abstract:**
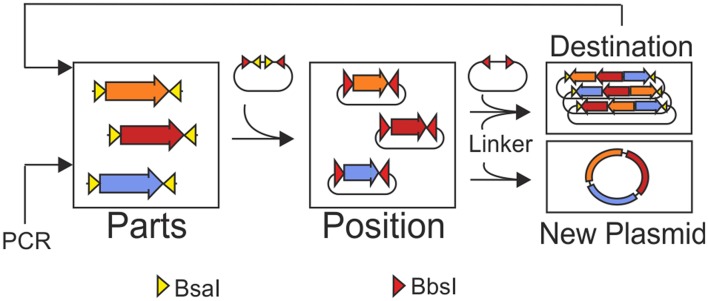
MoCloFlex allows storing genetic parts in different positions and combining these positions in any arrangement and order to custom plasmids. Flexibility is achieved by the help of linker plasmids added to the Golden Gate reaction. Moreover, MoCloFlex allows for iterative and flexible assembly of large constructs.

## 1. Introduction

Cloning is the fundamental method of molecular biology. Independent of the topic, most experiments start with cloning of plasmids or larger constructs. Cloning comprises adding of a tag, engineering a genetic circuit, or even building large constructs, like synthetic chromosomes. Thereby, parts of the cloning work are frequently used, thinking of antibiotic cassettes, origins of replication, tags or reporters. Hence, a modular, cloning approach can save time and resources. Identifying the needs for systematic cloning, the synthetic biology community has become one of the main drivers in the development of novel cloning systems (Ellis et al., 2011). In parts, this is achieved by hierarchical cloning systems, which promote standardization in the whole field of cloning. Methods like Golden Gate cloning and its descendant Modular Cloning (MoClo), reduce the needs for different restriction enzymes by building their systems around type IIs restriction enzymes (Engler et al., 2008; Weber et al., 2011; Sarrion-Perdigones et al., 2013; Casini et al., 2015). Since type IIs restriction enzymes cut outside their recognition site, they allow a rational design of overhangs. Additionally, using type IIs restriction enzymes allow arranging their restriction sides in such a way, they get lost when a cut vector and the insert ligate. Thus, restriction and ligation can be performed in a single step, making purification and a separate ligation step obsolete. During this so-called one-pot reaction, the final product gets enriched over time (Engler et al., 2008; Weber et al., 2011). Following the idea of standardized cloning, a variety of methods appeared in the last years (Casini et al., 2015). This list gets added on frequently, e.g., by EcoFlex, MODAL, PODAC, etc. Storch et al. (2015); Moore et al. (2016); Van Hove et al. (2017). Recent adaptations of the modular cloning standard are, e.g., dealing with the construction of synthetic chromosomes (Messerschmidt et al., 2016; Schindler et al., 2016; Zumkeller et al., 2018) or adding libraries of parts for a specific model organism (Lee et al., 2015; Moore et al., 2016; Rajkumar et al., 2019). Altogether, those adaptations are building a broad cloning toolbox, that still is growing.

**Figure 1:**
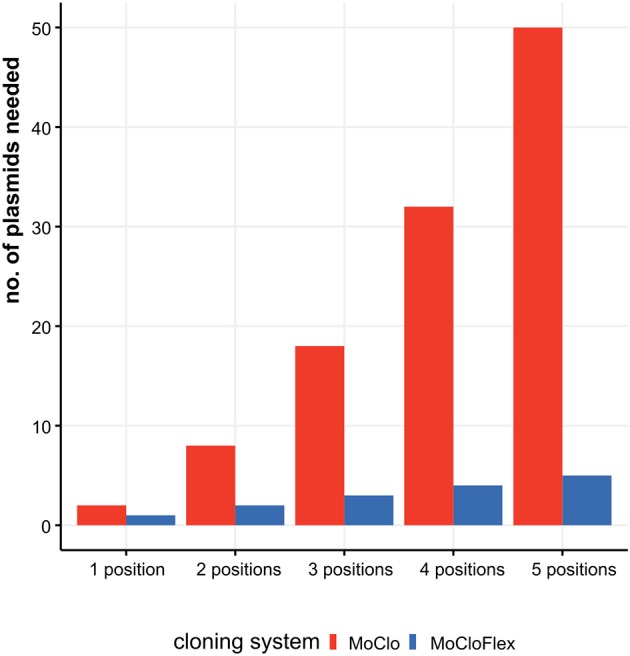
Construction effort needed to test combinatorial assemblies ranging from one to five fragments with MoClo standard compared to MoCloFlex. Testing genetic parts systematically mean to investigate every part at every position and in each orientation. Using MoClo the number of plasmids to built is *n* = *parts* * *positions* * *orientations*. Compared to that, MoCloFlex needs *n* = *positions* plasmids. With MoClo (red bars) the number of plasmids grows to up to 50 plasmids for testing five positions, whereas MoCloFlex (blue bars) only needs 5 plasmids for the same experiment.

Thereby, almost all modular cloning systems focus on building gene arrangements from small parts like promoters, ribosome binding sites, coding sequences, and terminators to bigger networks. In the logic of modular cloning systems, the storage plasmid defines the position of a DNA fragment in the next level. In the MoClo standard, the level 0 plasmids store the basic parts. Level 0 plasmids can be combined and have a fixed destination stored in level 1 plasmids. Those level 1 plasmids, usually storing transcription units, can be assembled in the same way as level 0 plasmids, to networks or chromosomes (level 2) (Weber et al., 2011; Engler et al., 2014; Messerschmidt et al., 2016; Schindler et al., 2016). This hierarchical structure of exiting modular system is, on the one side a straight forward and automatable process, on the other side leads to inflexibility and massive cloning effort if, e.g., transcription units shall be tested in different arrangements (Figure 1). Only one method allows to freely combine DNA-units in a smart one-level approach by changing the restriction-ligation protocol, adding a step, where linkers have to be fused to the fragments (Casini et al., 2013; Storch et al., 2015). Nevertheless, a method that allows to freely combine DNA-units and thereby is compatible with the MoClo standard is missing.

Here, we present a set of plasmids which adds a flexible path to Modular Cloning and also opens a way to build plasmids *de novo* in a modular way. The plasmid set comprises five position-vectors to store any DNA-fragment, 60 pre-built linker plasmids that allow combining two to five position vectors in any orientation and order which can significantly reduce cloning effort (Figure 1). The combination of position vectors can form a new plasmid or built into a destination vector. Our destination vector allows iterative rounds of cloning. Iterative cloning in our system needs an extra cloning step from the MCF-Destination into any MCF-Position vector from this one can fill another MCF-Destination. By the cost of an extra cloning step, the flexibility of the system is conserved into the next iterations, and no additional linkers or plasmids are needed. In the case that after some iterations, no more flexibility is needed, one can quickly transfer the construct stored in the MCF-Destination vector into the level 1 plasmids of the MoClo system and use the iterations described by Weber et al. (2011).

Since MoCloFlex is compatible with the Modular Cloning standard by Weber et al. (2011), a user can take an existing level 0 MoClo library to build transcription units into MoCloFlex position vectors. The other way around, a network made with MoCloFlex can be built into a level 1 plasmid of the MoClo plasmid set. Testing our system, we built one to five position vectors into a destination vector in different arrangements. Furthermore, we used our system to create a customized plasmid out of 3 position vectors.

## 2. Materials and Methods

### 2.1. Plate Fluorometry Assays

To test the efficiency of MoCloFlex, fluorometric assays were performed using an Infinite® 200 PRO plate reader (Tecan). Therefore, plasmids were transformed into chemical competent *E. coli* strain Top10 (Green and Rogers, 2013) and incubated over night on LB plates containing chloramphenicol (17.5 μg/mL) for selection. From this plates 94 clones were selected and inoculated into 150 μL fresh LB medium. Cells were grown for 16 h at 37°C and cultures were shook at 200 rpm. Afterwards, OD (600 nm) and fluorescence of mCherry (ex: 587 nm, em: 610 nm) (Shu et al., 2006), mVenus (ex: 515 nm, em: 527 nm) (Kremers et al., 2006), and mTurqouise2 (ex: 434 nm, em: 474 nm) (Goedhart et al., 2012) was measured. Data analysis was done using R Studio RStudio Team (2015) and plots were generated using the ggplot2 package (Wickham, 2016). The efficiency of MoCloFlex plasmid construction was performed using the same assay but with chemical competent MG1655 and MG1655::I-SceI (this work) instead (**Table 2**).

### 2.2. Chemicals, Oligonucleotides, and Reagents

Restriction enzymes, Desoxinucleotides, T4 DNA Ligase (conc.), 1 kb DNA Ladder and Phusion DNA Polymerase were purchased from New England BioLabs (Frankfurt, Germany). Oligonucleotides were purchased from Sigma-Aldrich (Steinheim, Germany). For DNA-Preparations Rotiprep® kits from Carl Roth GmbH + Co. KG were used (Karlsruhe, Germany). Chemicals were purchased from Carl Roth GmbH + Co. KG (Karlsruhe, Germany). Sequencing was performed by GATC Biotech AG (Constance, Germany) and Seqlab–Sequence Laboratories Göttingen GmbH (Göttingen, Germnay).

### 2.3. Plasmids

All plasmids, that were used or built during this work can be found in Table S1. Also in Table S1 is a PCR-template for building parts into MCF-Position vectors. All plasmids used in this work are available for the scientific community and can be requested from the authors. A .zip archive containing all plasmid maps as .gb is in the supplements (Data Sheet 3).

### 2.4. One-Pot Assembly

The restriction-ligation process was performed as described in Weber et al. (2011) with the concentrations given in Table 1.

**Table 1:** MoCloFlex one pot reaction mixture.

**Component**	**Stock conc**.	**Volume**	**End conc**.
T4 Ligase Buffer	10x	2.5 μL	1x
Restriction enzyme (BsaI-HF, BbsI)	10 U/μL	0.5 μL	0.2 U
T4 Ligase	2, 000 U/μL	0.5 μL	40 U/μL
each MCF-position vector			40 fmol
each MCF-Linker			40 fmol
MCF-backbone			40 fmol
H_2_O		ad 25 μL	

### 2.5. Strains

All plasmids, that carry the *ccdB* gene were build using DB3.1 cells (Invitrogen).

### 2.6. Media and Culture Conditions

*E. coli* cells were grown in Lysogeny Broth (LB) with 1 % tryptone, 0.5 % yeast extract and 1 % sodium chloride. Solid culture medium (LA) was made adding 12 g/L agar-agar to LB medium. If required, LB and LA were supplemented with antibiotics at given concentrations: ampicillin (100 μg/mL), streptomycin (100 μg/mL), kanamycin (50 μg/mL) and chloramphenicol (35 μg/mL). Liquid cultures shook at 200 rpm and 37°C for all experiments.

## 3. Results and Discussion

### 3.1. Modular and Flexible Cloning—MoCloFlex

**Figure 2:**
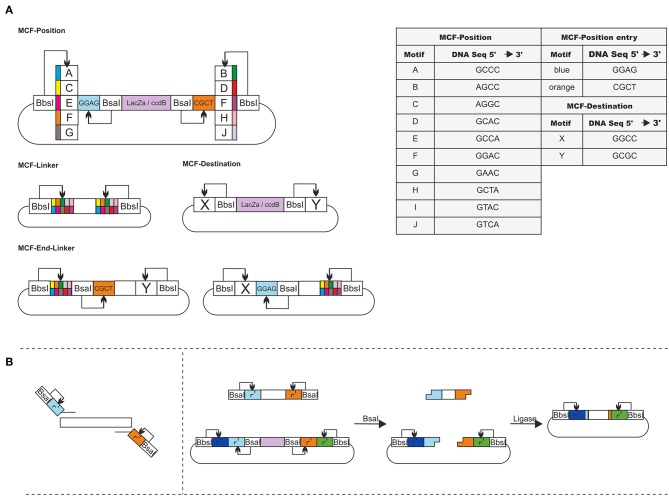
**(A)** Overview of MoCloFlex: MCF-Positions contain an inner insertion cassette accessible through insertion motifs (blue and orange; BsaI). The insertion cassette is flanked by combination motifs (A–J; BbsI). Every MCF-Linker has one combination of two motifs A–J. MCF-End-Linker contain one destination motif (X or Y; BbsI) to any combination motif (A–J) and have one integration motif. MCF-Destination plasmids have a *ccdB* and *lacZ* gene for selection flanked by the destination motifs X and Y (BbsI). **(B)** Any DNA-fragment that shall be inserted into an MCF-Position needs to get the insertion motifs by PCR. When cutting with BsaI and ligated with each another, both MCF-Position and fragment lose their BsaI recognition sites, and the fragment replaces the integration box of the MCF-Position.

The idea of MoCloFlex is to maximize flexibility when building gene arrangements, and at the same time, to allow standardization of the parts in such a way they can integrate into the existing MoClo standard. To achieve this, MoCloFlex comprises four plasmid classes: (1) MCF-Positions: can store DNA-fragments, either obtained and modified by PCR or through an existing MoClo Level 0 library. (2) MCF-Linkers: connect the different MCF-Positions to higher-order arrangements and mediate flexibility. (3) MCF-End-Linkers: allow building arrangements of MCF-Positions into the (4) MCF-Destination vector (Figure 2A). There are two steps to build a DNA-fragment into the MCF-Position. Sequences and functional parts of MCF-Linker, MCF-End-Linker and MCF-Positions can be found in Table S1. First, a PCR is necessary to add a BsaI cut side and the entry motifs (GGAG and CTCG) to the fragment. Whatever fragment shall be built into an MCF-Position has to be free of BsaI, and BbsI recognition sites. The entry motifs are the same as for level 1 plasmids of the MoClo standard, which realize compatibility between MoClo and MoCloFlex. Second, the PCR-fragment and the MCF-Position can be assembled by one-pot restriction-ligation (Figure 2B). Note, that it takes 24 h to obtain the final plasmid since the additional step of sequencing can be omitted due to the fact, that no further PCR is done between the construction of the parts and the final assembly. Together, restriction-ligation (5:00 h), transformation (1:30 h), plating and incubation (overnight, 8:00 h), colony PCR (1:45 h) growing right clones in liquid culture (6:00 h), and plasmid prep (1:45 h) could be done in one day.

**Figure 3:**
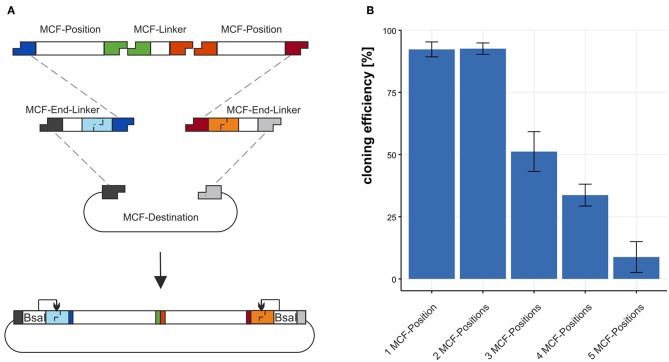
Using the MCF-Destination to build arrangements of MCF-Positions. **(A)** To build MCF-Positions into the MCF-Destination vector MCF-End-Linkers fuse MCF-Positions with the MCF-Destination vector and MCF-Linkers fuse MCF-Positions. **(B)** The cloning efficiency of 1 to 5 MCF-Positions (4–12 DNA-fragments) built into the MCF-destination vector. We define efficiency in % as positive clones per tested clones. Error bars represent standard deviation from 3 individual experiments with 95 clones each.

**Figure 4:**
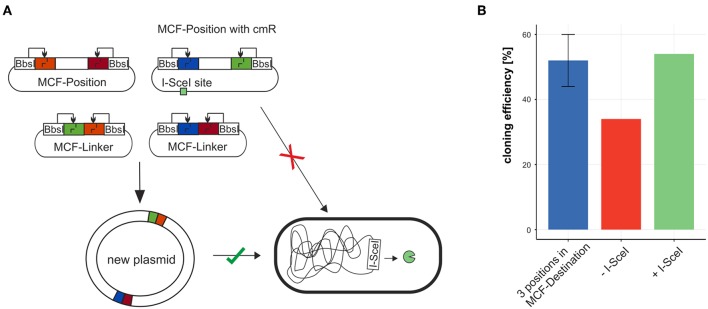
Combining MCF-Positions and MCF-Linkers to a new plasmid. **(A)** The plasmid carrying the antibiotic resistance marker cmR cannot be maintained in a strain expressing I-SceI thus reducing background colonies and restoring cloning efficiency **(B)**.

MCF-Positions and MCF-Linkers can be used to build plasmids by storing all parts of the plasmid on different MCF-Position vectors and connect them with MCF-Linker (**Figure 4A**). To build, e.g., networks or circuits of transcription units into the MCF-Destination vector, MCF-Linker and MCF-End-Linkers connect MCF-positions, with the MCF-Destination vector and add the entry motifs again, allowing rounds of iterative cloning. 40 MCF-Linkers and 20 MCF-End-Linker allow combining any MCF-Position motifs with each other and with the MCF-Destination vector. In Figure S1 the process is explained with a minimal example guiding through the planning of a plasmid and the selection of MCF-parts to the one-pot reaction. The system allows to build up to five MCF-Positions at the same time into the MCF-Destination vector (Figure 3A). To avoid errors by mixing the systems, we chose only motifs for the restriction overhangs, that are not part of the MoClo system, except for the entry motifs into the MCF-Positions, which are necessary to mediate compatibility. As another feature, the overhangs are prefix and suffix free concerning BsaI and BbsI (BpiI) recognition sites, which excludes an accidental formation of new recognition sites and therefore a failure of the assembly (Figure 2A, table).

### 3.2. Efficiency of Cloning MCF-Positions Into the MCF-Destination Vector

We tested the efficiency of the restriction-ligation reaction from the minimal amount of 4 DNA-fragments to a maximum of 12 DNA-fragments in one reaction. We build three MCF-positions each with a different fluorescence protein regulated by an *aldA* promoter and two “dummy”-positions containing sequence without biological function. To test the efficiency 1 to 5 MCF-Positions were assembled into the MCF-Destination vector and the fraction of positive clones was determined. For the first construct 1 MCF-Position, we combined 4 DNA-fragments in the restriction-ligation: The MCF-Destination, two MCF-End-Linkers (XA and BY) and the MCF-Position AB. Each additional MCF-Position also needs another MCF-Linker; thus, two additional MCF-Linker vectors must be added to the reaction for every additional MCF-Position in the restriction-ligation. We calculate the efficiency from clones that display the expected fluorescence as a fraction of total colonies on the plates for the test of 10 and 12 DNA-fragments sequencing was used to confirm the results. For 4 DNA-fragments (1 MCF-Position) the efficiency is 92.3 ± 3 %, for 6 DNA-fragments (2 MCF-Positions) in the restriction-ligation the efficiency is 92.6 ± 2.3 % for 8 DNA-fragments (3 MCF-Positions) it is dropping to 51.2 ± 8.1 % and when using 10 DNA-fragments (4 MCF-Positions) it is still at 33.7 ± 4.5 %. The maximum we built with our system is 12 DNA-fragments (5 MCF-Positions) in one reaction, with an efficiency of 8.8 ± 6.2 % (Figure 3B). Notably, the number of colonies was also dropping from 2021 colonies with 4 fragments to 189 with 12 fragments/reaction. Since the constructs we chose contained highly repetitive sequences such as mTurquiose2, mVenus, mCherry, and three times the same promoter and terminator sequences, which make a construct difficult to clone, cloning efficiency is likely to be higher when using MoCloFlex with non-repetitive sequences.

### 3.3. Cloning Efficiency of *de novo* Plasmid Assembly

To test the efficiency of building plasmids with MoCloFlex, using only MCF-Positions and MCF-Linkers, we created a plasmid out of 6 MCF-Parts: MCF-Position AB contained an origin of replication (P15A), MCF-Position CD contained a chloramphenicol resistance cassette, MCF-Position EF contained a CDS of mCherry, and 3 MCF-Linker (BC, DE, FA) bridging between the MCF-Positions. When we first built plasmids using this setup, background colonies that carry the uncut MCF-Position with the chloramphenicol resistance cassette appeared, which reduced the efficiency from 50 % to around 35 % (Figure 4B). To solve that problem we integrated an I-SceI recognition site (Monteilhet et al., 1990) onto the MCF-Position CD and transformed our constructs into an *E. coli* strain that is expressing I-SceI meganuclease (Table 2) (Monteilhet et al., 1990), this avoids cloning background with uncut MCF-Position plasmids (Figure 4B) and restored efficiency to the 50 % expected when using 6 fragments in one reaction.

**Table 2:** Strains used in this work.

**Strain**	**Description**	**References**
DB3.1	F-, *gyrA*462, *endA*1, glnV44, Δ(*sr1-recA*), mcrB, mrr, hsdS20(rB-, mB-), ara14, galK2, lacY1, proA2, rpsL20(Smr), xyl5, Δleu, mtl1	Bernard et al., 1993
Top10	F- *mcrA* Δ (mrr-hsdRMS-mcrBC) Φ 80lacZΔM15 Δ lacX74 nupG recA1 araD139 Δ (ara-leu)7697, galE15, galK16 rpsL(StrR), endA1 λ-	Invitrogen
MG1655	K-12 F- λ ilvG rfb-50 rph-1	Blattner et al., 1997
MG1655Δ*LacIZYA*::*I-SceI*	Δ(*lac*)::*I-SceI*	this work
Top10Δ*insPinsQ*::*I-SceI*	Δ*insPinsQ*::*I-SceI*	this work
DB3.1Δ*insPinsQ*::*I-SceI*	Δ*insPinsQ*::*I-SceI*	this work

### 3.4. RecBCD Digestion Increases Cloning Efficiency but Decreases Number Clones

The sequencing of negative clones revealed recombination events, that could not be explained by the *in vitro* restriction-ligation reaction. Hence, the recombination took place *in vivo*. To test whether partially assembled linear fragments recombined *in vivo* after transformation, we incubated the reaction after the restriction-ligation with RecBCD. RecBCD is an enzyme complex that, in *E. coli* takes part in homologous recombination but also has a nuclease function for double-strand and single-strand DNA (Amundsen et al., 1986; Yu et al., 1998). RecBCD incubation previous to transformation of a 8 DNA-fragment assembly (3 MCF-Positions) decreased the number of colonies from around 250 to 10 but also increased the cloning efficiency dramatically from 50 to 90 %. Inferring from these preliminary results, we think that ligation or recombination of linear DNA-fragments occurs to a significant level after transformation. The capability of *E. coli* to make plasmids out of double stranded DNA is used by a couple of *in vivo* cloning methods, e.g., Beyer et al. (2015) and the mechanism is shown to be dependent on the exonuclease III XthA (Nozaki and Niki, 2019) which is present in our cloning strain so maybe the unwanted recombination is due to this mechanism. The mechanism is dependent on homologous sequences which can be found in the fluorescent proteins, the promoters, and the terminators used in this study. Nevertheless, after RecBCD digestion colonies only appeared with a maximum of 8 fragments in the restriction-ligation and thus is not applicable for improving the cloning efficiency of more than 3 MCF-Positions in one reaction.

### 3.5. The Arrangement Influences the Expression of Transcription Units in a Network

**Figure 5:**
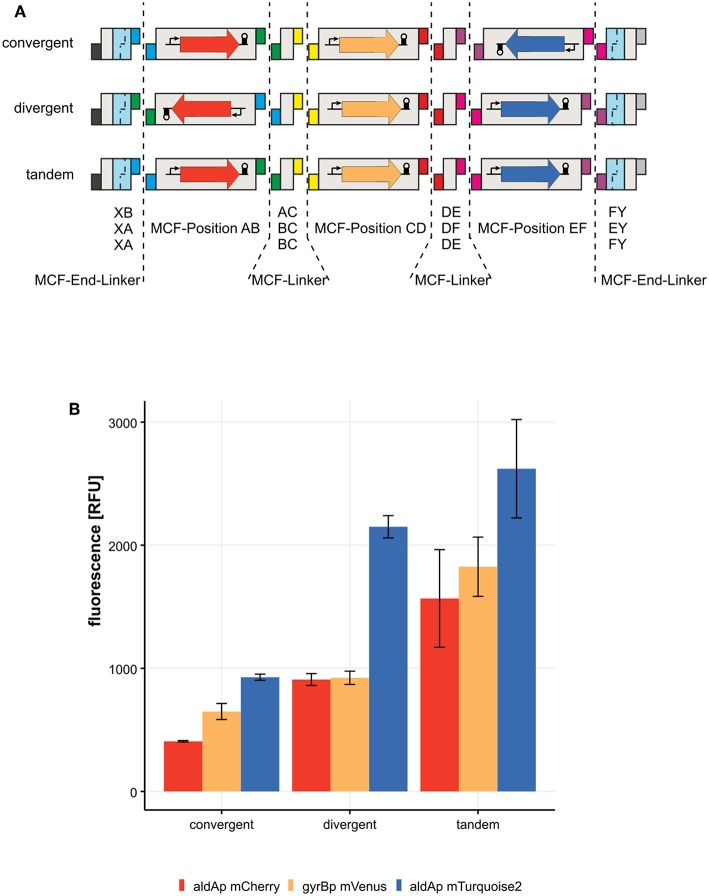
Expression of three transcription units in three different contexts. **(A)** list of MCF-Linkers, MCF-End-Linkers and MCF-Positions, that are built into the MCF-Destination vector. Convergent: transcription from *aldAp* controlling mCherry and *aldAp* controlling mTurquiose2 expression, pointing toward *gyrBp* controlling mVenus expression. Divergent: *aldAp* promoters pointing away from *gyrBp*. Tandem: every transcription unit points into the same direction. **(B)** Expression in [RFU] of the three transcription units in the three different arrangements.

To test the flexibility of MoCloFlex, we decided to build three networks of three different transcription units in three arrangements. The first transcription unit consists of *gyrBp* controlling the expression of mVenus and a T0 terminator. The other two transcription units are either mCherry or mTurquoise2 controlled by *aldAp* and a T0 terminator. In the first construct, both flanking transcription units directed to the *gyrBp* controlled transcription unit, which is called a convergent arrangement. Second, we arranged both *aldAp* controlled transcription units in a divergent orientation in respect to the *gyrBp* cassette. In the last orientation, all cassettes point in the same direction, which is called tandem orientation (Figure 5A). As shown in Figure 5B, we observed differential expression patterns in all three arrangements with a maximal expression for all three transcription units when in tandem orientation. The arrangements are on plasmids maintained by a P15A origin of replication which is closely related to the ColE1 origin of replication (Selzer et al., 1983). The P15A origin leads to around 10 copies of the plasmid per cell. Read-through transcription can interfere with the replication initiation in ColE1 (Stueber and Bujard, 1982). Inferring from this, it could be that different transcription arrangements lead to slightly different copy numbers changing the expression level. However, our transcription units harbor terminators protecting the origin of replication in every tested arrangement. As another possibility, the plasmid topology could be altered by the arrangement of three transcription units, since almost half of the *E. coli* promoters, including the *gyrA* promoter, are responding to altered DNA supercoiling either induced globally or by neighboring expression (Lim et al., 2003; Sobetzko, 2016; Dages et al., 2018). It could be that the differences in expression levels are due to *aldA* and *gyrB* promoters respond to different DNA supercoiling levels they get from their neighbors, but this should be investigated further in a chromosomal context. However, there is a measurable alteration in the expression levels of our three constructs, and this is dependent on the arrangement of the transcription units, which confirms the need for testing arrangements systematically. Hence, arrangement and orientation matters and can easily be screened with MoCloFlex.

### 3.6. Conclusion

We present MoCloFlex, a new modular cloning system for flexible *de novo* part/plasmid assembly. It provides significant advantages to classical cloning concerning efficiency, modularity and number of fragments. Therefore, MoCloFlex combines the advantages of established modular systems regarding flexibility and cloning effort. Moreover, we focus on compatibility and standardization of present systems and therefore considered compatibility with widely used modular cloning systems. MoCloFlex consists of a small set of flexible position plasmids and a fixed set of linker plasmids. Once plasmids are isolated and stocked, a single one-pot cloning step is needed to combine any arrangement, orientation and number of parts to a new part or even complete plasmid. Our system, therefore, allows for the synthesis of custom made parts in any combination up to small synthetic chromosomes. We have shown that a custom plasmid can be planned, built and isolated within 24 h. The reusability of parts reduces space consumption for cloning significantly as all custom plasmids can be synthesized from a small set of plasmids. Its high flexibility and secure handling also considers the need for everyday dynamic adaptations of plasmids during research, leading to reduced research costs and time consumption. With the presented advantages and compatibility to the previous standard Modular Cloning, MoCloFlex contributes to the implementation of this cloning standard in the field of synthetic biology. Together with MoCloFlex, this standard can now flexibly design and assemble parts, plasmids and synthetic chromosomes.

## Data Availability Statement

The datasets generated for this study are available on request to the corresponding author.

## Author Contributions

CK and PS designed the method and wrote the article. CK, LE, and AK performed the experiments.

### Conflict of Interest

The authors declare that the research was conducted in the absence of any commercial or financial relationships that could be construed as a potential conflict of interest.
