# Supplementary material for: MoCloFlex: A Modular Yet Flexible Cloning System
Source: Front Bioeng Biotechnol. 2019 Oct 17;7:271. doi: 10.3389/fbioe.2019.00271 (PMC6843054; doi:10.3389/fbioe.2019.00271)
Supplement: Supplementary file 1 [file Data_Sheet_1.pdf]

Table S1

Plasmid name	info	antibiotic resistance	entry restriction enzyme	release restriction enzyme
MCF_EndLinker_AY		Spectinomycin	BbsI	BsaI
MCF_EndLinker_BY		Spectinomycin	BbsI	BsaI
MCF_EndLinker_CY		Spectinomycin	BbsI	BsaI
MCF_EndLinker_DY		Spectinomycin	BbsI	BsaI
MCF_EndLinker_EY		Spectinomycin	BbsI	BsaI
MCF_EndLinker_FY		Spectinomycin	BbsI	BsaI
MCF_EndLinker_GY		Spectinomycin	BbsI	BsaI
MCF_EndLinker_HY		Spectinomycin	BbsI	BsaI
MCF_EndLinker_IY		Spectinomycin	BbsI	BsaI
MCF_EndLinker_JY		Spectinomycin	BbsI	BsaI
MCF_EndLinker_XA		Spectinomycin	BbsI	BsaI
MCF_EndLinker_XB		Spectinomycin	BbsI	BsaI
MCF_EndLinker_XC		Spectinomycin	BbsI	BsaI
MCF_EndLinker_XD		Spectinomycin	BbsI	BsaI
MCF_EndLinker_XE		Spectinomycin	BbsI	BsaI
MCF_EndLinker_XF		Spectinomycin	BbsI	BsaI
MCF_EndLinker_XG		Spectinomycin	BbsI	BsaI
MCF_EndLinker_XH		Spectinomycin	BbsI	BsaI
MCF_EndLinker_XI		Spectinomycin	BbsI	BsaI
MCF_EndLinker_XJ		Spectinomycin	BbsI	BsaI
MCF_Linker_AD		Spectinomycin	BbsI	
MCF_Linker_AE		Spectinomycin	BbsI	
MCF_Linker_AG		Spectinomycin	BbsI	
MCF_Linker_AJ		Spectinomycin	BbsI	
MCF_Linker_BC		Spectinomycin	BbsI	
MCF_Linker_BD		Spectinomycin	BbsI	
MCF_Linker_BE		Spectinomycin	BbsI	
MCF_Linker_BF		Spectinomycin	BbsI	
MCF_Linker_BG		Spectinomycin	BbsI	
MCF_Linker_BH		Spectinomycin	BbsI	
MCF_Linker_BI		Spectinomycin	BbsI	
MCF_Linker_BJ		Spectinomycin	BbsI	
MCF_Linker_CA		Spectinomycin	BbsI	
MCF_Linker_CE		Spectinomycin	BbsI	
MCF_Linker_CF		Spectinomycin	BbsI	
MCF_Linker_CG		Spectinomycin	BbsI	
MCF_Linker_CH		Spectinomycin	BbsI	
MCF_Linker_CI		Spectinomycin	BbsI	
MCF_Linker_CJ		Spectinomycin	BbsI	
MCF_Linker_DE		Spectinomycin	BbsI	
MCF_Linker_DF		Spectinomycin	BbsI	
MCF_Linker_DG		Spectinomycin	BbsI	
MCF_Linker_DH		Spectinomycin	BbsI	
MCF_Linker_DI		Spectinomycin	BbsI	
MCF_Linker_DJ		Spectinomycin	BbsI	
MCF_Linker_EG		Spectinomycin	BbsI	

MCF_Linker_EH		Spectinomycin	BbsI	
MCF_Linker_EI		Spectinomycin	BbsI	
MCF_Linker_FA		Spectinomycin	BbsI	
MCF_Linker_FG		Spectinomycin	BbsI	
MCF_Linker_FH		Spectinomycin	BbsI	
MCF_Linker_FI		Spectinomycin	BbsI	
MCF_Linker_FJ		Spectinomycin	BbsI	
MCF_Linker_GJ		Spectinomycin	BbsI	
MCF_Linker_HA		Spectinomycin	BbsI	
MCF_Linker_HI		Spectinomycin	BbsI	
MCF_Linker_HJ		Spectinomycin	BbsI	
MCF_Linker_IA		Spectinomycin	BbsI	
MCF_Linker_IG		Spectinomycin	BbsI	
MCF_Linker_JE		Spectinomycin	BbsI	
MCF_Position_AB	I-SceI-site	Kanamycin	BsaI	BbsI
MCF_Position_AB_dummy		Kanamycin		BbsI
MCF_Position_AB_p15A		Kanamycin		BbsI
MCF_Position_CD	I-SceI-site	Kanamycin	BsaI	BbsI
MCF_Position_CD_cmR		Kanamycin; Chloramphenicol		BbsI
MCF_Position_CD_cmR_I-SceI	I-SceI-site	Kanamycin; Chloramphenicol		BbsI
MCF_Position_CD_dummy		Kanamycin		BbsI
MCF_Position_CD_I-SceI	I-SceI site	Kanamycin	BsaI	BbsI
MCF_Position_EF	I-SceI-site	Kanamycin	BsaI	BbsI
MCF_Position_EF_dummy		Kanamycin		BbsI
MCF_Position_EF_mC		Kanamycin		BbsI
MCF_Position_GH	I-SceI-site	Kanamycin	BsaI	BbsI
MCF_Position_GH_mV		Kanamycin		BbsI
MCF_Position_IJ	I-SceI-site	Kanamycin	BsaI	BbsI
MCF_Position_IJ_mTq2		Kanamycin		BbsI
MCF-Destination_XY		Chloramphenicol	BsaI	
pLE17_gyrBp_mC_mV_mTq_MCFbb_convergent		Chloramphenicol		BsaI
pLE18_gyrBp_mC_mV_mTq_MCFbb_divergent		Chloramphenicol		BsaI
pLE51_gyrBp_mC_mV_mTq_MCFbb_tandem		Chloramphenicol		BsaI

"Functional" parts of the MoCloFlex plasmids

MCF-End-Linker

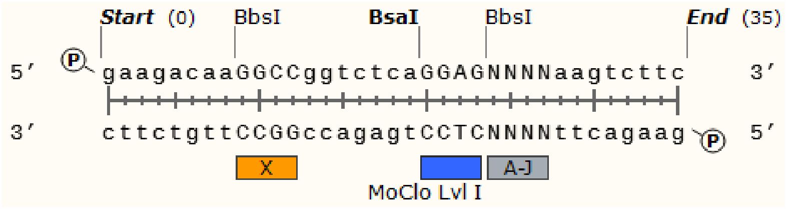

MCF-Linker

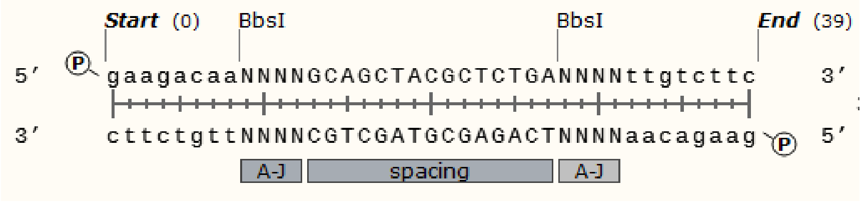

MCF-Positions

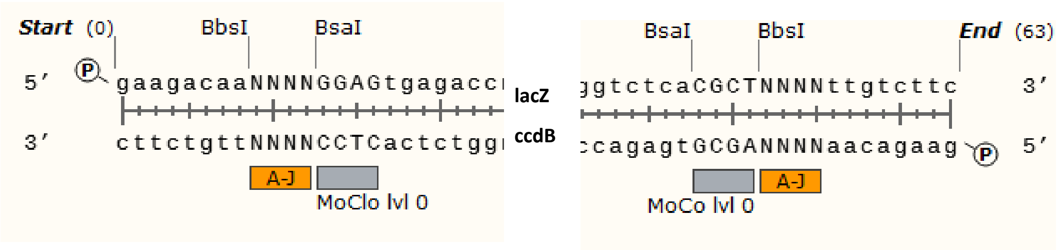

fw Primer 3' extension

Cgggtctca GGAG Sequence to integrate into MCF-Position

rev Primer 5' extension

Gcggtctca AGCG Sequence to integrate into MCF-Position

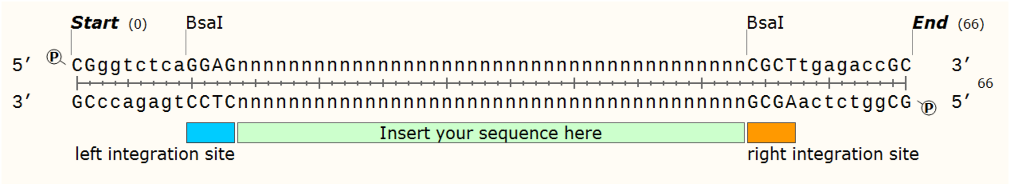

made with SnapGene
