# Supplementary material for: MoCloFlex: A Modular Yet Flexible Cloning System
Source: Front Bioeng Biotechnol. 2019 Oct 17;7:271. doi: 10.3389/fbioe.2019.00271 (PMC6843054; doi:10.3389/fbioe.2019.00271)
Supplement: Supplementary file 2 [file Data_Sheet_2.PDF]

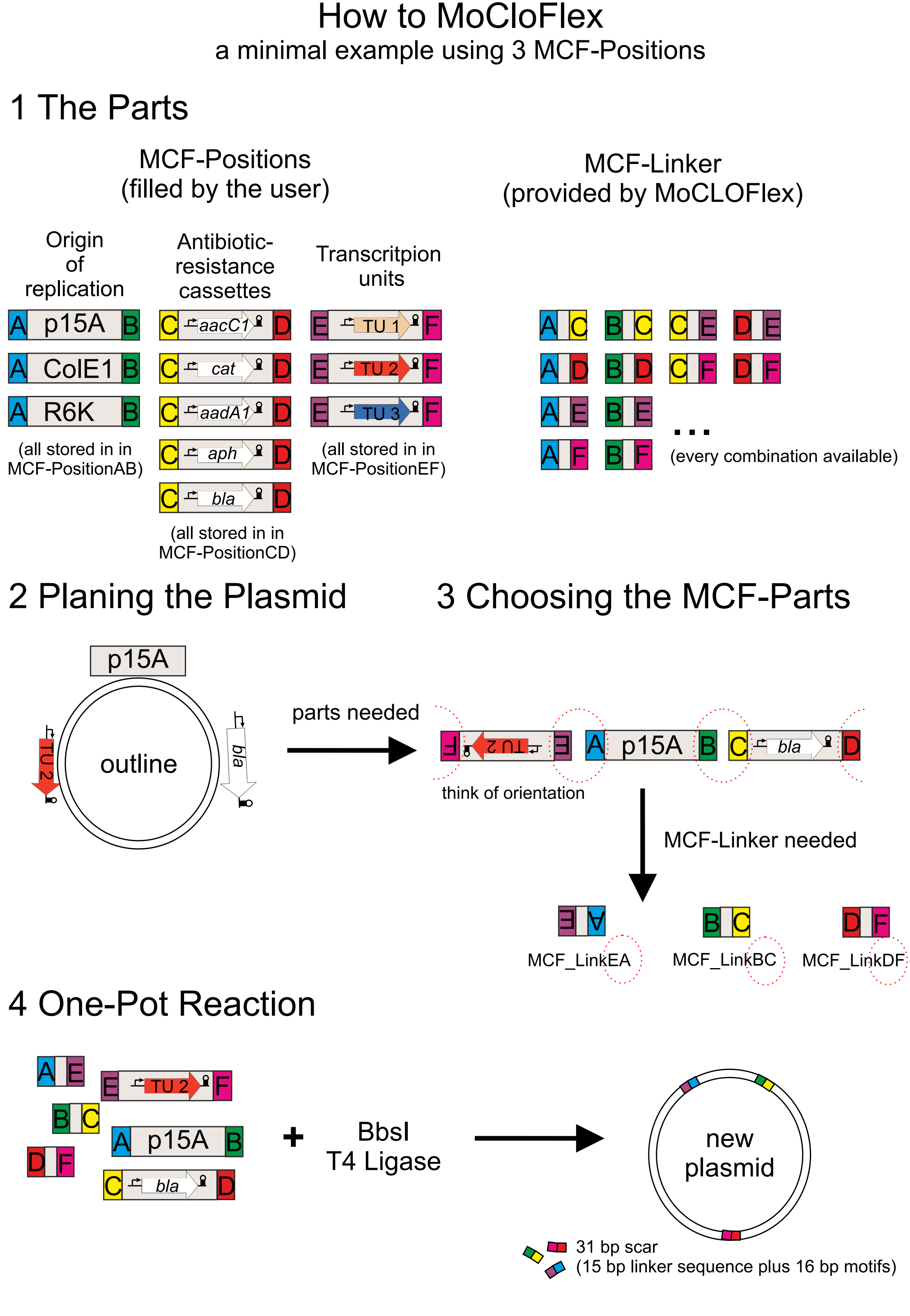

Figure S1. A step by step manual on how to plan and built a plasmid with MoCloFlex. In this example, we have a minimal example of a genetic parts library 3 of 5 possible positions are in use. In MCF-PositionAB different origin of replication, in MCF- PositionCD antibiotic resistance cassettes, and in MCF-PositonEF transcription units are stored. The MCF-Linker bridging all motifs are prebuilt and come with MoCloFlex. Making such a library is the first step and is described in the main article. The second step is to outline how the plasmid should look in the end. Here, the mid-copy ori p15A flanked by an ampicillin resistance cassette and a transcription unit, both pointing away from the ori, shall be built. In the next step, the MCF-Positions can be linearly displayed in the arrangement from the outline using a cloning software of your choice. Take care of the wanted orientations. Now, the motifs that flank the MCF-Positions have to be linked by MCF-Linkers. There are linkers for every combination pick the ones that have both motifs to be bridged in their name. The last step is the One-Pot Reaction, as described in the main article.
